# Reduced nitric oxide bioavailability impairs myocardial oxygen balance during exercise in swine with multiple risk factors

**DOI:** 10.1007/s00395-021-00890-8

**Published:** 2021-08-26

**Authors:** Jens van de Wouw, Oana Sorop, Ruben W. A. van Drie, Jaap A. Joles, A. H. Jan Danser, Marianne C. Verhaar, Daphne Merkus, Dirk J. Duncker

**Affiliations:** 1 Department of Cardiology, Division of Experimental Cardiology, Erasmus University Medical Center, PO Box 2040, 3000 CA Rotterdam, Netherlands; 2 Department of Nephrology and Hypertension, University Medical Center Utrecht, Utrecht, Netherlands; 3 Department of Internal Medicine, Erasmus MC University Medical Center, Rotterdam, Netherlands; 4 Walter Brendel Center of Experimental Medicine (WBex), University Clinic Munich, 81377 LMU Munich, Germany; 5 German Center for Cardiovascular Research (DZHK), Munich Heart Alliance (MHA), Partner Site Munich, 81377 Munich, Germany

**Keywords:** Diabetes mellitus, Chronic kidney disease, Coronary blood flow, Coronary microcirculation, Coronary microvascular dysfunction

## Abstract

In the present study, we tested the hypothesis that multiple risk factors, including diabetes mellitus (DM), dyslipidaemia and chronic kidney disease (CKD) result in a loss of nitric oxide (NO) signalling, thereby contributing to coronary microvascular dysfunction. Risk factors were induced in 12 female swine by intravenous streptozotocin injections (DM), a high fat diet (HFD) and renal artery embolization (CKD). Female healthy swine (*n* = 13) on normal diet served as controls (Normal). After 5 months, swine were chronically instrumented and studied at rest and during exercise. DM + HFD + CKD swine demonstrated significant hyperglycaemia, dyslipidaemia and impaired kidney function compared to Normal swine. These risk factors were accompanied by coronary microvascular endothelial dysfunction both in vivo and in isolated small arteries, due to a reduced NO bioavailability, associated with perturbations in myocardial oxygen balance at rest and during exercise. NO synthase inhibition caused coronary microvascular constriction in exercising Normal swine, but had no effect in DM + HFD + CKD animals, while inhibition of phosphodiesterase 5 produced similar vasodilator responses in both groups, indicating that loss of NO bioavailability was principally responsible for the observed coronary microvascular dysfunction. This was associated with an increase in myocardial 8-isoprostane levels and a decrease in antioxidant capacity, while antioxidants restored the vasodilation to bradykinin in isolated coronary small arteries, suggesting that oxidative stress was principally responsible for the reduced NO bioavailability. In conclusion, five months of combined exposure to DM + HFD + CKD produces coronary endothelial dysfunction due to impaired NO bioavailability, resulting in impaired myocardial perfusion at rest and during exercise.

**Supplementary Information:**

The online version contains supplementary material available at 10.1007/s00395-021-00890-8.

## Introduction

The prevalence of classic risk factors for cardiovascular disease, such as obesity, diabetes mellitus (DM), dyslipidaemia, and chronic kidney disease (CKD), is predicted to increase in the coming years [29, 74]. It is well established that these risk factors can lead to coronary atherosclerosis and its sequelae including angina pectoris and myocardial infarction [18]. However, a significant number of patients with risk factors, especially women, have angina without any signs of significant coronary artery stenosis on coronary angiography [1, 8, 10, 45, 57, 58], which has been termed “ischemia and no obstructive coronary artery disease” (INOCA) [1, 45]. INOCA is a multifactorial syndrome of angina that is associated with a variety of risk factors, in which coronary microvascular dysfunction plays a central role [1, 8, 10]. Interestingly, coronary microvascular dysfunction is not only a common denominator in INOCA, diastolic dysfunction and heart failure with preserved ejection fraction, but also predicts future hospitalization for heart failure with preserved ejection fraction [21, 66]. Furthermore, patients with INOCA show an elevated risk of cardiovascular events including stroke and acute coronary syndrome [1, 8, 20, 42, 57, 66]. These observations demonstrate the pathophysiological as well as prognostic importance of coronary microvascular dysfunction in several cardiovascular disease entities, suggesting that coronary microvascular dysfunction represents an important therapeutic target [1, 8, 10]. However, other than treating risk factors, there is still no effective therapy for INOCA, which appears to be due to an incomplete understanding of the underlying pathophysiology and a limited number of appropriate translational animal models for INOCA [63].

Recently, we reported that chronic exposure of female swine to a combination of DM, a high fat diet (HFD) and CKD (DM + HFD + CKD) resulted in reductions in coronary flow reserve and perturbations in myocardial perfusion and oxygen delivery with evidence of myocardial anaerobic metabolism, reminiscent of INOCA [71]. These abnormalities were associated with reduced myocardial capillary density, increased interstitial fibrosis, and left ventricular diastolic dysfunction [60] and, importantly, occurred in the absence of epicardial coronary atherosclerosis, indicative of coronary microvascular dysfunction. The latter did not appear to be the result of structural alterations of coronary resistance vessels, as arteriolar density and arteriolar wall to lumen ratio were maintained, suggesting that impaired coronary microvascular tone control—likely involving endothelial dysfunction—was responsible [71].

The mechanism underlying the impaired control of coronary microvascular tone in DM + HFD + CKD swine was not assessed in our previous study [71]. Understanding the mechanisms underlying coronary microvascular dysfunction is critical to develop novel targeted therapies of INOCA [1, 8, 10, 45]. Evidence is increasing that endothelial dysfunction plays a key role in coronary microvascular dysfunction [1, 46, 63]. Hence, we hypothesized that a loss of NO-cGMP signalling, was principally responsible for the observed impaired regulation of coronary microvascular tone in our swine model [71]. To test this hypothesis, we subjected female swine to either 5 months of DM + HFD + CKD or control conditions and—using pharmacological agents to interrogate the NO-cGMP signalling pathway—we performed (i) in vivo studies in chronically instrumented swine, under awake resting conditions and during graded treadmill exercise, followed by (ii) in vitro studies in isolated porcine coronary small arteries. Additionally, we performed (iii) left ventricular myocardial tissue analysis to explore the molecular basis for the alterations in NO-cGMP signalling.

## Materials and methods

### Animals

All animal experiments were approved by the Animal Care Committee at the Erasmus University Medical Center (Rotterdam, The Netherlands, AVD1010020185224) and in accordance with the guidelines from Directive 2010/63/EU of the European Parliament on the protection of animals used for scientific purposes. Twelve Yorkshire x landrace female swine (24 ± 1 kg) were included in the DM + HFD + CKD group, while 13 healthy female swine of similar age and weight served as control group (Normal). In the DM + HFD + CKD group, one animal died after CKD induction and one animal died shortly after instrumentation. In the Normal swine group, one animal died due to technical complications during surgical instrumentation. An overview of the experimental timeline is presented in Supplementary Fig. 1.

### Induction of risk factors

The induction of risk factors in the DM + HFD + CKD group has been described in detail elsewhere [60]. Briefly, DM was produced by injection of streptozotocin (50 mg^−1^ kg^−1^ day^−1^ IV on 3 consecutive days, AdipoGen Life Sciences, San Diego, CA, USA) [60]. The severity and stability of DM was monitored bi-weekly by measurements of blood glucose and ketone levels. Two weeks after DM induction, animals were sedated with an intramuscular injection of a cocktail of Zoletil (tiletamine/zolazepam; 5 mg kg^−1^), Sedazine (xylazine; 2.25 mg kg^−1^) and atropine (2 mg), intubated and artificially ventilated with a mixture of O_2_ and N_2_ (1:2 vol/vol), to which 1–2% (vol/vol) isoflurane was added for anaesthesia. CKD was produced by global micro-embolization of the right kidney as well as regional micro-embolization of the lower pole of the left kidney. For this purpose, the renal arteries, (right renal artery and the arterial branch perfusing the left lower renal pole) were catheterized under fluoroscopy guidance with a Swan-Ganz catheter, inserted through a 9F sheath in the right common carotid artery. Following inflation of the balloon to prevent back-flow into the aorta, 75 mg of polyethylene microspheres with a diameter of 38–42 μm (Cospheric, Santa Barbara, CA) were infused into each renal artery [60]. The wound was closed and the animals were allowed to recover. One week after CKD induction, an isocaloric high fat diet containing 10% sucrose, 15% fructose, 25% saturated fats and 1% cholesterol (Research Diet Services B.V., Wijk bij Duurstede, The Netherlands) supplemented with sodium chloride (20 g day^−1^) was gradually introduced [60]. The Normal group continued to receive regular swine chow. Animals were housed in pairs but were fed separately and had ad libitum access to drinking water.

### Surgical instrumentation

The details of the surgical instrumentation have been described elsewhere [12, 71]. Briefly, after an overnight fast, Normal and DM + HFD + CKD swine (5 months after CKD induction) were sedated with an intramuscular injection of a cocktail of Zoletil (tiletamine/zolazepam; 5 mg kg^−1^), Sedazine (xylazine; 2.25 mg kg^−1^) and atropine (2 mg), intubated and artificially ventilated with O_2_ and N_2_ (1:2; vol/vol), to which 2–3% (vol/vol) isoflurane was added for maintenance of anaesthesia. Under sterile conditions, fluid-filled polyvinylchloride catheters were placed in the pulmonary artery for infusion of drugs and in the left ventricle (LV) and aorta for pressure measurements and arterial blood sampling. A transonic flow probe (Transonic Systems Inc., Ithaca, NY, USA) was placed around the proximal left anterior descending coronary artery for measurement of coronary blood flow. Finally, two small angio-catheters (one as back-up) were inserted into the anterior inter-ventricular vein for sampling of coronary venous blood from the left anterior descending artery perfusion territory. Electrical wires and catheters were tunnelled subcutaneously to exit at the back and protected with a vest. Then, the chest was closed and animals were allowed to recover, receiving analgesia (0.3 mg buprenorphine IM) and a slow-release fentanyl patch (50 μg h^−1^) for 3 days, as well as antibiotic prophylaxis (2000 mg amoxicillin IV) for 7 days. All catheters were flushed daily with heparinized saline (1000–5000 IU ml^−1^ saline) to prevent the formation of blood clots and maintain catheter patency. In the DM + HFD + CKD group, two animals had a malfunctioning coronary flow probe and were, therefore, not included in the exercise experiments. In the Normal group, in one animal, the coronary venous angio-catheters lost patency prior to the exercise studies and one animal was unable to exercise due to lameness following instrumentation.

### Resting studies

#### Glomerular filtration rate

The glomerular filtration rate (GFR) was measured in awake, resting swine using continuous inulin infusion (19 mg min^−1^, Inutest, Fresenius Pharma Kabi Austria GmbH, Graz, Austria) and plasma sampling [60, 71]. Three consecutive 20-min clearance periods were averaged.

#### Coronary vasodilator function in vivo

Coronary microvascular endothelium-independent vasodilation was assessed using the NO-donor sodium nitroprusside (SNP, Sigma-Aldrich, Saint Louis, MO, USA) and coronary microvascular endothelium-dependent vasodilation was assessed using adenosine triphosphate (ATP, Sigma-Aldrich). With swine resting quietly, heart rate, mean aortic blood pressure and coronary blood flow were recorded at baseline and during consecutive 10-min infusions of SNP (0.5–5.0 µg kg^−1^ min^−1^) and ATP (50–300 µg kg^−1^ min^−1^). Haemodynamic variables were recorded after 8-min of infusion of each dose, under steady-state conditions.

#### Coronary flow reserve (CFR)

On a different day, coronary blood flow was measured in awake resting swine under basal conditions and during maximal coronary vasodilation using IV infusion of adenosine (0.5 mg kg^−1^ min^−1^, Sigma-Aldrich), in combination with phenylephrine (5–7.5 µg kg^−1^ min^−1^) to maintain mean arterial pressure at baseline levels [61, 71]. CFR was calculated as the ratio of maximal coronary blood flow and basal coronary blood flow.

### Exercise studies

One to two weeks after instrumentation, experiments were conducted, with swine exercising on a motor-driven treadmill [71]. Animals underwent three exercise trials, each performed on a separate day and in random order. For this purpose, resting haemodynamic measurements, blood samples, and rectal temperature were obtained with swine standing quietly on the treadmill. Subsequently, swine were subjected to a three-stage incremental treadmill exercise protocol (2, 3 and 4 km h^−1^ at 0% inclination, 3 min per stage). Haemodynamic variables were continuously recorded digitally on a Codas workstation (ATCODAS, Dataq Instruments, Akron, OH, USA) with blood samples collected during the final 30 s of each 3-min exercise stage when steady-state haemodynamics had been achieved. Blood samples were analyzed for partial oxygen pressure (pO_2_), haemoglobin oxygen saturation (SaO_2_), and haemoglobin concentration (ABL-800, Radiometer, Copenhagen, Denmark).

On a different day, the exercise experiments were repeated in the presence of NO synthase (NOS) inhibition by IV infusion of 20 mg kg^−1^ of N_ω_-Nitro-L-arginine (NLA, Sigma-Aldrich). Fifteen min after NLA administration, resting samples were obtained and the exercise protocol was performed as described above.

On another day, animals were studied in the presence of phosphodiesterase 5 (PDE5) inhibition, by 10 mg of Sildenafil (Revatio, Pfizer Inc, New York, NY, USA) infused IV over a 10-min period; 5 min after completion of administration, resting samples were obtained and the exercise protocol repeated.

### Termination

At termination, swine were sedated by intramuscular injection with a cocktail of Zoletil (tiletamine/zolazepam; 5 mg kg^−1^), Sedazine (xylazine; 2.25 mg kg^−1^) and atropine (2 mg) and anesthetized with pentobarbital (9 mg kg^−1^ h^−1^ IV), intubated and artificially ventilated with O_2_ and N_2_ (1:2; vol/vol), to which 2–2.5% (vol/vol) isoflurane was added. Subsequently, a sternotomy was performed, ventricular fibrillation was induced using a 9 Volt battery, and the heart was immediately excised and weighed. Left ventricular tissue samples were prepared and stored for later biochemical and molecular analyses, and coronary small arteries were isolated for in vitro functional experiments.

### Coronary small artery function in vitro

Coronary microvascular endothelium-dependent and -independent vasodilator responses were assessed in coronary small arteries (∼300 µm diameter) isolated from the epicardial surface of the left ventricular apex and studied in vitro using a Mulvany wire myograph (Danish Myograph Technology, Aarhus, Denmark) [64, 72]. Following preconstriction with 10^−6^ mol L^−1^ thromboxane-A2 analogue U46619 (Sigma–Aldrich), concentration–response curves were determined for the endothelium-independent NO-donor *S*-nitroso-*N*-acetylpenicillamine (SNAP, 10^−10^ to 10^−5^ mol L^−1^, Sigma–Aldrich), and for the endothelium-dependent vasodilator bradykinin (BK, 10^−10^ to 10^−6^ mol L^−1^, Sigma–Aldrich) in the absence and presence of NOS blockade with 10^–4^ mol L^−1^ Nω-Nitro-L-arginine methyl ester hydrochloride (LNAME, Sigma-Aldrich), the highly water soluble prodrug of NLA [48]. In a subgroup of animals (4 Normal and 5 DM + HFD + CKD swine), the concentration-response curves for bradykinin were also performed in the absence and presence of the ROS scavenger *N*-2-mercaptopropionylglycine (MPG, 10^−5^ mol L^−1^, Sigma–Aldrich) and the superoxide dismutase mimetic 4-hydroxy-2,2,6,6-tetramethylpiperidine-*N*-oxyl (Tempol 10^−3^ mol L^−1^, Sigma–Aldrich).

### Measurements in plasma

Fasting arterial blood samples were obtained at the time of instrumentation (5 months follow-up) for determination of plasma glucose, triglycerides, total cholesterol, low-density lipoprotein (LDL), high-density lipoprotein (HDL), albumin and creatinine. Arterial plasma concentrations of tumor necrosis factor alpha (TNF-α) and NO metabolites nitrite and nitrate (NO_2_^−^ + NO_3_^−^) were determined using a ELISA kit (R&D Systems Inc., Minneapolis, MN, USA) and a colorimetric Griess reaction assay (Biovision Inc., Milpitas, CA, USA), respectively, according to the manufacturer’s instructions. sNOX2-dp measurement (E13651327, Sincere Bio, Beijing China) was performed in undiluted coronary venous plasma of both Normal and DM + HFD + CKD animals according to the manufacturer’s instructions.

### Left ventricular tissue analysis

#### Western blots

Total endothelial NOS (eNOS), phosphorylated eNOS (Ser1177 and Thr495 sites), eNOS S-gluthathionylation, eNOS monomer and dimer protein levels as well as vasodilator-stimulated phosphoprotein (VASP) and phosphorylated VASP (p-VASP) were determined in frozen, homogenized bulk subendocardial left ventricular tissue samples. SDS-PAGE for phosphorylated eNOS, total eNOS protein content, VASP and phosphorylated VASP, and housekeeping protein β-actin was performed at room temperature. For detection of eNOS monomer and dimer fractions, low-temperature SDS-PAGE in the absence of β-mercaptoethanol was performed as previously described [40, 60]. Briefly, gels and buffers were equilibrated at 4 °C before electrophoresis, and the buffer tank was placed in an ice bath during electrophoresis to maintain a low temperature.

Following SDS-PAGE, the proteins were transferred to nitrocellulose membranes and the blots were probed with primary anti-phospho eNOS Ser1177 (1:1000, purified monoclonal rabbit anti-human eNOS, CST9570, Cell Signalling Technology Inc., Danvers, MA, USA), anti-phospho eNOS Thr495 (1:1000, purified polyclonal rabbit anti-human eNOS, CST9574, Cell Signalling Technology Inc.), anti-eNOS (1:500, purified monoclonal Mouse anti-human eNOS, 610,297, Transduction Laboratory, BD Biosciences, San Jose, CA, USA), anti-VASP (1:1000, purified monoclonal rabbit anti-human VASP, CST3132, Cell Signalling Technology Inc.), anti-phospho VASP Ser239 (1:1000, purified polyclonal rabbit anti-human VASP, CST3114, Cell Signalling Technology Inc.) and anti-β-actin (1:1000 rabbit monoclonal antibody CST4970, Cell Signalling Technology Inc.). All blots were analyzed using the Odyssey CLX system (LI-COR; Thermo Fisher Scientific, Waltham, MA, USA). S‐glutathionylation of eNOS was determined by immunoprecipitation of left ventricular homogenates. Briefly, 50 µg Dynabeads (Protein G Immunoprecipitation Kit, 10007D, Thermo Fisher Scientific) coupled to 5 µg anti-eNOS antibody (purified monoclonal Mouse anti-human eNOS, 610,297, BD Transduction Laboratories) were incubated for 30 min at room temperature under constant shaking with 100 µl 15 µg protein µl^−1^ endocardial left ventricular homogenate. After washing, protein was eluded from the Dynabead complex and blotted as described above. Purified monoclonal Mouse IgG1 κ was used as isotype control (554,121, BD Biosciences). Blots were probed with a mouse anti-human glutathione monoclonal antibody (1:1000, MA1-7620, Invitrogen) and a rabbit anti-human polyclonal eNOS antibody (1:1000, 9572, Cell Signalling Technology Inc.). Quantification was done using the LI-COR Odyssey system as described above.

#### Reverse transcription qPCR

Gene expression of nicotinamide adenine dinucleotide phosphate oxidase (NADPH oxidase 2, NOX2), phosphodiesterase 5 (PDE5) and eNOS (NOS3) was measured in myocardial tissue. Total RNA was isolated from bulk left ventricular subendocardial frozen samples via homogenisation of small pieces of tissue (< 30 mg) by adding RLY lysisbuffer (Bioline, London, United Kingdom) and 2-mercaptoethanol (Sigma-Aldrich) using a homogenizer. After proteinase K (Invitrogen, Carlsbad, CA, USA) treatment at 55 °C for 10 min, total RNA was isolated using the ISOLATE II RNA Mini Kit (Bioline). RNA purity and concentration were measured and cDNA synthesis (SensiFAST cDNA synthesis kit, Bioline) was performed using 500 ng RNA as input. Gene expression was analyzed on the CFX96 Real-Time PCR detection system (Bio-rad, Hercules, CA, USA) using the SensiMix SYBR-green supermix (Bioline). Primers used are shown in Supplementary Table 1. Results were normalized to the housekeeping genes hypoxanthine phosphoribosyltransferase 1 (HPRT1) and  Ribosomal Protein L13 (RPL13) as previously described [44]. Relative changes in expression levels were calculated using the BioRad CFX software.

#### NO metabolites, total antioxidant capacity, 8-isoprostane and PDE5 activity measurements

Snap frozen bulk subendocardial left ventricular samples were used for measuring the myocardial levels of the NO metabolites nitrite and nitrate (NO_2_^−^ + NO_3_^−^), by a colorimetric Griess reaction assay (Biovision Inc., Milpitas, CA, USA), the total myocardial antioxidant capacity (Total Antioxidant Capacity Assay kit, Abcam plc., Cambridge, United Kingdom), and total 8-isoprostane (Cayman Chemicals, Ann Arbor, MI, USA). Overall phosphodiesterase and phosphodiesterase 5 activity in the myocardium was measured using a IMAP assay (PDE(5) IMAP FP PDE evaluation kit, Molecular Devices, San José, CA, USA).

### Data analysis and statistics

Data from 13 Normal swine and 11 DM + HFD + CKD swine were included for analysis. It should be noted that, while all animals but two (one in each group), were also included in our previous study [71], all Tables and Figures presented here contain new and original data not previously published. Only in Table 1 did we include functional data of those animals that were also part of our previous study, for the purpose of describing the baseline phenotype of animals included in the Normal versus DM + HFD + CKD group.

**Table 1:** Phenotypical aspects of Normal and DM + HFD + CKD swine

	Normal (*n* = 13)	DM + HFD + CKD (*n* = 11)
Body weight (kg)	99 ± 5	103 ± 5
Metabolic function		
Plasma fasting glucose (mmol L^−1^)	8.8 ± 0.6	19.9 ± 1.3*
Plasma total cholesterol (mmol L^−1^)	1.7 ± 0.1	9.5 ± 1.4*
Plasma LDL cholesterol (mmol L^−1^)	1.0 ± 0.2	7.9 ± 1.2*
Plasma HDL cholesterol (mmol L^−1^)	0.9 ± 0.1	2.7 ± 0.5*
Plasma LDL/HDL cholesterol ratio	1.2 ± 0.1	3.4 ± 0.5*
Plasma triglycerides (mmol L^−1^)	0.19 ± 0.02	0.33 ± 0.05*
Renal function		
Glomerular filtration rate (ml min^−1^)^a^	196 ± 11	129 ± 12*
Plasma creatinine (µmol L^−1^)	122 ± 4	169 ± 9*
Inflammation		
TNF-α (pg ml^−1^)^b^	26 ± 4	56 ± 5*
Plasma albumin (g L^−1^)	40 ± 1	31 ± 2*
Coronary microvascular function		
Coronary flow reserve^c^	3.6 ± 0.2	2.5 ± 0.2*

Values are mean ± SEM

*LDL* low-density lipoprotein, *HDL* high-density lipoprotein, *TNF-α* tumor necrosis factor alpha

*P < 0.05 for DM + HFD + CKD versus Normal by unpaired Students *t* test

^a^*n* = 9 Normal and *n* = 9 DM + HFD + CKD

^b^*n* = 10 Normal

^c^*n* = 4 Normal and *n* = 6 DM + HFD + CKD

Digital recording and offline analysis of haemodynamic data obtained at rest and during exercise have been described in detail elsewhere [2]. In short, myocardial O_2_ delivery (MDO_2_) was computed as the product of coronary blood flow and arterial blood O_2_ content, myocardial O_2_ consumption (MVO_2_) was calculated as the product of coronary blood flow and the difference in O_2_ content between arterial and coronary venous (cv) blood, and myocardial oxygen extraction (MEO_2_) was computed as 100% • MVO_2_/MDO_2_. The rate-pressure product (RPP) was computed as the product of heart rate and systolic aortic blood pressure. Coronary vascular conductance (CVC) was computed as coronary blood flow/mean aortic pressure (MAP). Coronary blood flow, MDO_2_, MVO_2_, and CVC were normalized per gram (g) of myocardium perfused by the left anterior descending coronary artery, which was estimated to be 40% of the left ventricle [71].

Statistical analysis was performed using SPSS Statistics 21.0 (IBM Corp, Armonk, NY, USA). Comparison of single time point variables between the two groups was performed by unpaired Student’s *t* test. In vitro coronary microvascular responses to pharmacological agents were analyzed using two-way ANOVA for repeated measures. Comparisons of correlations between eNOS protein and NO_2_^−^ + NO_3_^−^ measurements and of vascular responses to SNP/ATP, and SNAP/bradykinin were performed by logistic regression analysis. In vivo haemodynamic and myocardial oxygen balance responses to exercise and intervention were tested using three-way followed by two-way ANOVA and ANCOVA for repeated measures, followed by post hoc testing when appropriate, using least significant difference correction. Correlations were calculated using Pearson’s correlation coefficient. Statistical significance was accepted when *P* < 0.05 (two-tailed). A value of *P* < 0.10 (two-tailed) was considered a statistical trend.

## Results

### Model characteristics

At 5-months follow-up, body weight was similar between Normal and DM + HFD + CKD swine, but DM + HFD + CKD swine had profound metabolic dysregulation as evidenced by the presence of hyperglycaemia and dyslipidaemia, including hypercholesterolemia, increased LDL/HDL ratio and hypertriglyceridemia (Table 1). Furthermore, DM + HFD + CKD animals showed CKD, evidenced by lower GFR and elevated plasma creatinine levels. These abnormalities were associated with increased TNF-α and lower plasma albumin values, indicating a systemic pro-inflammatory state. Additionally, CFR in awake resting conditions was reduced by 30% in DM + HFD + CKD swine, suggestive of coronary microvascular dysfunction (Table 1).

### Coronary vasodilator function

**Fig. 1:**
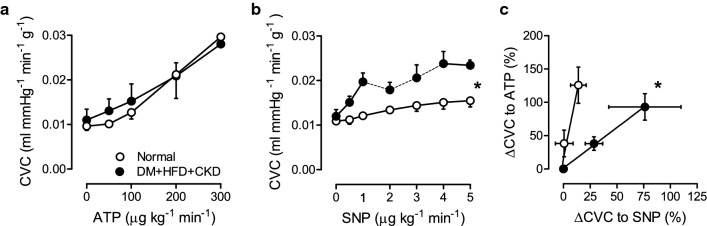
In vivo response of coronary vascular conductance (CVC) to infusion of SNP and ATP in Normal and DM + HFD + CKD swine. No differences in endothelium-dependent vasodilation to graded infusion of adenosine triphosphate (ATP) at rest, were observed in vivo (**a**). Endothelium-independent vasodilation to NO-donor sodium nitroprusside (SNP) was enhanced in DM + HFD + CKD swine compared to Normal (**b**) from 2 till 4 µg kg^−1^ min^−1^ we had to discontinue the experiment in 1 animal at each dose due to severe hypotension (< 40 mmHg), depicted by the dotted lines. There was a dissociation between endothelium-dependent (ATP 100–200 µg^−1^ kg^−1^ min^−1^) and endothelium-independent (SNP 0.5–1 µg^−1^ kg^−1^ min^−1^) vasodilation in DM + HFD + CKD compared to Normal swine, characterized by a clockwise rotation of the relation between the coronary vasodilator response to SNP versus ATP (**c**). Values are mean ± SEM. SNP: DM + HFD + CKD *n* = 5, Normal: *n* = 9 ATP: DM + HFD + CKD *n* = 4, Normal *n* = 8. *P < 0.05 for DM + HFD + CKD versus Normal by two-way ANOVA for repeated measures (**a** and **b**) and logistic regression analysis (**c**)

To assess endothelium-dependent and endothelium-independent vasodilator function in vivo, the changes in coronary vascular conductance in response to graded intravenous infusions of ATP and SNP were studied in resting Normal and DM + HFD + CKD swine. Infusion of ATP and SNP resulted in decreases in mean aortic pressure and—probably reflex-mediated—increases in heart rate in both Normal and DM + HFD + CKD swine (Supplementary Table 2). The haemodynamic responses to ATP were not different between the two groups, while the haemodynamic responses to NO-donor SNP were more pronounced in DM + HFD + CKD compared to normal swine. The endothelium-dependent vasodilator ATP produced dose-dependent coronary vasodilator responses that were similar in DM + HFD + CKD and Normal swine (Fig. 1a). However, DM + HFD + CKD animals showed an enhanced coronary vasodilator response to SNP (Fig. 1b), indicating increased coronary vascular smooth muscle sensitivity to NO. Since we have previously shown that ATP—in the doses used—produces coronary vasodilation that is essentially NO-mediated [16], the ATP- and SNP-responses could be interpreted to suggest that a reduced NO bioavailability was compensated for by increased coronary smooth muscle sensitivity to NO. This interpretation was further supported by the clockwise rotation of the relation between endothelium-dependent and endothelium-independent vasodilator responses in the DM + HFD + CKD compared to Normal swine (Fig. 1c).

**Fig. 2:**
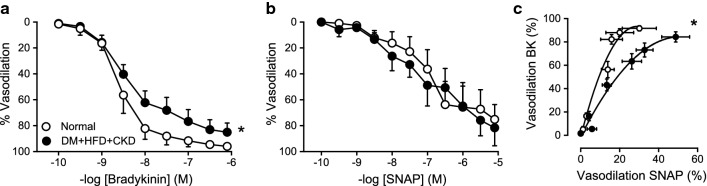
In vitro small coronary artery function in Normal and DM + HFD + CKD. In vitro endothelium-dependent vasodilation to bradykinin was blunted in DM + HFD + CKD compared to Normal swine (**a**), while endothelium-independent vasodilation to NO-donor (SNAP) was similar in both groups (**b**). Consequently, there was a clockwise rotation in the relation between the vasodilator responses to 10^–10^-10^–7^ mol L^−1^ bradykinin (BK) and SNAP 10^–10^-10^–7^ mol L^−1^ (**c**). Data are expressed as a percentage of the contraction induced by U46619. Values are mean ± SEM. Normal: BK *n* = 4, SNAP *n* = 5, DM + HFD + CKD: BK *n* = 8, SNAP *n* = 5. *P < 0.05 for DM + HFD + CKD versus Normal by two-way ANOVA for repeated measures (**a** and **b**) and logistic regression analysis (**c**)

Coronary small arteries, isolated from DM + HFD + CKD and Normal swine, and studied in vitro, showed similar preconstriction to 10^−6^ mol L^−1^ U46619 (i.e. 80 ± 11% vs. 79 ± 14% of the response to 10^−1^ mol L^−1^ KCl). Dose–response relaxation in response to the endothelium-dependent vasodilator bradykinin was significantly blunted in DM + HFD + CKD vessels (Fig. 2a) as compared to Normal, while the vascular smooth muscle responsiveness to NO appeared to be unchanged, as the response to the exogenous NO-donor SNAP was similar between groups (Fig. 2b). Importantly, and in line with the in vivo observations regarding SNP and ATP, the relationship between endothelium-dependent and -independent vasodilation in vitro was also rotated clockwise in DM + HFD + CKD swine, confirming endothelial dysfunction in the coronary small arteries of these animals (Fig. 2c).

**Fig. 3:**
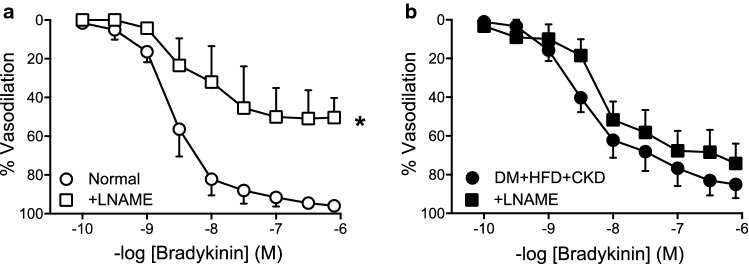
In vitro small coronary artery function in Normal and DM + HFD + CKD swine. NO synthase inhibition with LNAME attenuated endothelium-dependent vasodilation to bradykinin (BK) in Normal swine (**a**) while LNAME had no effect on BK-induced vasodilation in DM + HFD + CKD (**b**). Data are expressed as a percentage of the contraction induced by U46619. Values are mean ± SEM. Normal: BK and BK + LNAME *n* = 4; DM + HFD + CKD: BK and BK + LNAME *n* = 8. *P < 0.05 for effect LNAME by two-way ANOVA for repeated measures

Further experiments showed that NOS blockade by LNAME significantly attenuated the bradykinin-induced vasodilation (Fig. 3a) in Normal swine, while it had no effect on the vasodilator response to bradykinin in DM + HFD + CKD animals (Fig. 3b). These findings suggest that the observed coronary microvascular endothelial dysfunction was caused by a loss of NO bioavailability.

### Perturbations in myocardial oxygen balance during exercise: role of NO

**Fig. 4:**
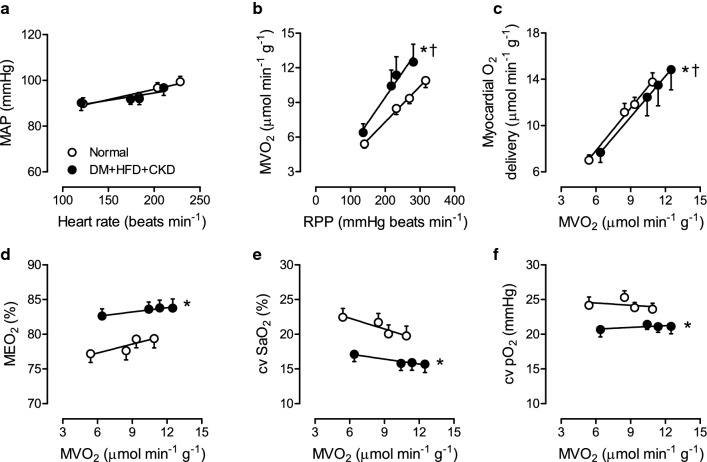
In vivo haemodynamics and myocardial oxygen balance at rest and during exercise in Normal and DM + HFD + CKD swine. DM + HFD + CKD and Normal swine showed similar relations between mean aortic pressure (MAP) and heart rate (**a**). DM + HFD + CKD had higher myocardial oxygen consumption (MVO_2_) for any given level of rate-pressure product (RPP, **b**). DM + HFD + CKD showed decreased myocardial oxygen delivery (**c**) especially during higher levels of MVO_2_, forcing an increase in  myocardial oxygen extraction (MEO_2_, **d**), resulting in lower coronary venous oxygen saturation (cv SaO_2_, **e**) and coronary venous partial oxygen pressure (cv pO_2_, **f**) compared to Normal. Values are mean ± SEM. DM + HFD + CKD: *n* = 8, Normal: *n* = 10. *P < 0.05 for DM + HFD + CKD versus Normal, ^†^*P* < 0.05 for interaction between MVO_2_ and group, by two-way ANCOVA for repeated measures

The impact of risk factors on myocardial oxygen balance was assessed at rest and during graded treadmill exercise (Fig. 4). The responses of heart rate and mean aortic pressure to exercise were slightly blunted in DM + HFD + CKD compared to Normal swine, but their relation remained unaffected (Fig. 4a). At any level of rate-pressure product (an index of myocardial oxygen demand) DM + HFD + CKD required higher levels of myocardial oxygen consumption compared to Normal swine, reflecting reduced myocardial efficiency (Fig. 4b). The higher levels of oxygen consumption were not fully met by a commensurate increase in myocardial oxygen delivery (Fig. 4c), necessitating an increase in myocardial oxygen extraction (Fig. 4d), that resulted in a decrease in coronary venous O_2_ saturation (Fig. 4e) and O_2_ tension (Fig. 4f). These findings indicate that the increased myocardial oxygen consumption in DM + HFD + CKD swine is not met by a commensurate increase in myocardial oxygen delivery due to perturbations in the regulation of coronary resistance vessel tone.

**Fig. 5:**
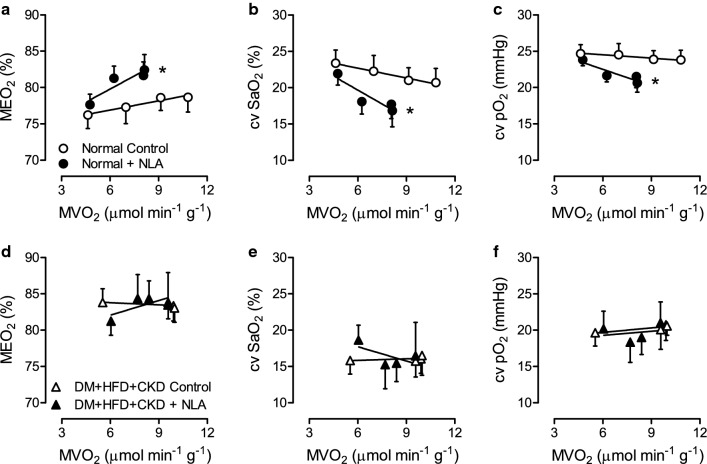
In vivo effect of NOS inhibition on myocardial oxygen balance at rest and during exercise in Normal and DM + HFD + CKD swine. Normal swine showed increased oxygen extraction (MEO_2_. **a**), lower coronary venous oxygen saturation (cv SaO_2_, **b**) and coronary venous partial oxygen pressure (cv pO_2_, **c**), while DM + HFD + CKD swine showed no change in oxygen extraction (**d**), coronary venous oxygen saturation (**e**) or coronary venous partial oxygen pressure (cv pO_2_, **f**) following the inhibition of NO synthase with N_ω_-Nitro-L-arginine (NLA) during exercise. Values are mean ± SEM. DM + HFD + CKD: *n* = 6, Normal: *n* = 7. *P < 0.05 versus corresponding Control by two-way ANCOVA for repeated measures

To evaluate whether alterations in endogenous NO production contributed to the perturbations in myocardial oxygen balance in DM + HFD + CKD swine, animals from both groups were subjected to treadmill exercise in the presence of NOS inhibition by NLA. Haemodynamic and myocardial oxygen balance data are shown in Table 2 and Fig. 5. NLA resulted in similarly robust increases in mean aortic pressure in both groups at rest and during exercise (Table 2), that were accompanied by reflex-mediated decreases in heart rate. NLA had negligible effects on arterial haemoglobin and arterial oxygen saturation levels in either group of swine (Table 2).

**Table 2:** Haemodynamic and myocardial oxygen balance during exercise with and without NOS inhibition in Normal and DM + HFD + CKD swine

	Group	Intervention	*n*	Standing	Exercise			ANOVA *P* value		
					2	3	4	NLA	Group	NLA *Group
Heart rate (beats min^−1^)	Normal	Control	9	117 ± 5	174 ± 11*	204 ± 12*	232 ± 8*			
		NLA	9	110 ± 9	143 ± 8*^‡^	167 ± 8*^‡^	196 ± 12*^‡^	< 0.001		
	DM + HFD + CKD	Control	8	115 ± 6	154 ± 7*	169 ± 10*^†^	190 ± 11*^†^		0.006	
		NLA	8	108 ± 8	148 ± 12*	152 ± 10*	153 ± 8*^‡†^	0.026	0.145	0.338
MAP (mmHg)	Normal	Control	9	90 ± 3	95 ± 3	99 ± 2*	101 ± 3*			
		NLA	9	116 ± 6^‡^	119 ± 5^‡^	119 ± 4^‡^	122 ± 3^‡^	< 0.001		
	DM + HFD + CKD	Control	8	86 ± 4	90 ± 3	92 ± 3	96 ± 4		0.017	
		NLA	8	116 ± 5^‡^	113 ± 5^‡^	118 ± 5^‡^	128 ± 3^‡^	< 0.001	0.682	0.085
CBF (ml min^−1^ g^−1^)	Normal	Control	7	1.01 ± 0.12	1.36 ± 0.16	1.66 ± 0.17*	1.91 ± 0.21*			
		NLA	7	1.07 ± 0.12	1.21 ± 0.13	1.40 ± 0.20	1.42 ± 0.17	0.201		
	DM + HFD + CKD	Control	6	1.22 ± 0.11	1.83 ± 0.23*	1.80 ± 0.27	1.81 ± 0.16*		0.109	
		NLA	6	1.43 ± 0.14	1.58 ± 0.11	1.59 ± 0.10	1.56 ± 0.13	0.406	0.004	0.458
Hemoglobin (g dl^−1^)	Normal	Control	7	9.4 ± 0.4	10.4 ± 0.6	10.9 ± 0.5*	10.9 ± 0.6*			
		NLA	7	8.9 ± 0.4	9.9 ± 0.7	11.2 ± 0.8*	10.6 ± 0.6*	0.332		
	DM + HFD + CKD	Control	6	8.6 ± 0.3	11.2 ± 0.7*	10.3 ± 0.5*	10.4 ± 0.5*		0.124	
		NLA	6	8.1 ± 1.1	9.5 ± 0.8	10.0 ± 1.2	11.5 ± 2.7	0.279	0.381	0.913
Arterial SaO_2_ (%)	Normal	Control	7	98 ± 1	98 ± 1	98 ± 1	98 ± 1			
		NLA	7	98 ± 1	97 ± 1	96 ± 1	97 ± 1	0.081		
	DM + HFD + CKD	Control	6	98 ± 1	97 ± 1	98 ± 1	98 ± 1		0.875	
		NLA	6	99 ± 1	97 ± 1	98 ± 1	99 ± 1	0.031	0.027	0.044
CV SaO_2_ (%)	Normal	Control	7	23.4 ± 1.8	22.3 ± 2.2	21.0 ± 1.5	20.7 ± 2.0			
		NLA	7	21.9 ± 1.5	18.1 ± 1.6	16.8 ± 2.0	17.7 ± 1.7	0.018		
	DM + HFD + CKD	Control	6	15.8 ± 1.9^†^	16.1 ± 2.0	15.8 ± 2.2	16.5 ± 1.9		< 0.001	
		NLA	6	18.6 ± 2.0	15.3 ± 3.3	15.5 ± 2.5	16.5 ± 4.6	0.634	0.124	0.092
CV pO_2_ (mmHg)	Normal	Control	7	24.7 ± 1.3	24.5 ± 1.5	23.9 ± 1.2	23.8 ± 1.3			
		NLA	7	23.9 ± 0.8	21.6 ± 0.8	20.6 ± 1.3	21.5 ± 1.5	0.006		
	DM + HFD + CKD	Control	6	19.6 ± 1.8^†^	20.7 ± 2.1	20.1 ± 2.7	20.6 ± 1.3		0.001	
		NLA	6	20.2 ± 2.4	18.4 ± 2.8	19.0 ± 2.4	21.0 ± 2.9	0.647	0.076	0.335
MVO_2_ (µmol min^−1^ g^−1^)	Normal	Control	7	4.6 ± 0.6	7.0 ± 1.0	9.1 ± 1.0*	10.8 ± 1.3*			
		NLA	7	4.8 ± 0.6	6.2 ± 1.0	8.1 ± 1.3*	8.1 ± 1.2*	0.376		
	DM + HFD + CKD	Control	6	5.5 ± 0.7	9.9 ± 1.6*	9.6 ± 1.4*	10.0 ± 1.4*		0.215	
		NLA	6	6.0 ± 0.9	7.7 ± 0.6	8.4 ± 1.0	9.6 ± 2.4	0.507	0.081	0.691

Values are mean ± SEM

*MAP* mean arterial pressure, *CBF* coronary blood flow per gram of myocardium, *SaO*_*2*_ oxygen saturation, *CV* coronary venous, *pO*_*2*_ partial oxygen pressure, *MVO*_*2*_ myocardial oxygen consumption per gram of myocardium, *NLA* N_ω_-Nitro-L-arginine (20 mg kg^−1^ IV)

*P < 0.05 versus corresponding Standing

^†^*P* < 0.05 versus corresponding Normal

^‡^*P* < 0.05 versus corresponding Control by two-way ANOVA for repeated measures and post hoc analysis with least significant difference correction

In Normal swine, NOS inhibition resulted in an increase in myocardial oxygen extraction with consequent decreases in coronary venous O_2_ saturation and O_2_ tension, particularly during exercise (Fig. 5a–c), indicating that endogenous NO exerted a small vasodilator influence and contributed to exercise-induced dilation of coronary resistance vessels of healthy swine. In contrast, NLA had no discernible effect on myocardial oxygen extraction, coronary venous O_2_ saturation or O_2_ tension in DM + HFD + CKD swine plotted either as a function of myocardial oxygen consumption (Fig. 5d–f) or as a function of the rate-pressure product, (Supplementary Fig. 2), an index of myocardial oxygen demand that is mathematically unrelated to myocardial oxygen extraction, coronary venous O_2_ saturation and O_2_ pressure [26, 69]. These findings indicate that the bioavailability and coronary vasodilator influence of endogenous NO was lost in DM + HFD + CKD animals. Loss of NO in the coronary microcirculation of swine with DM + HFD + CKD is further supported by the observation that in the presence of NOS inhibition, the levels of myocardial oxygen extraction and coronary venous O_2_ saturation and O_2_ tension were no longer different between Normal and DM + HFD + CKD swine (Supplementary Fig. 3 and Table 2). This loss of contribution of endogenous NO to the regulation of coronary resistance vessel tone in exercising swine with risk factors is consistent with the in vitro experiments that demonstrated a similar loss of contribution of NO to the vasodilator responses to bradykinin.

### Role of PDE5

**Fig. 6:**
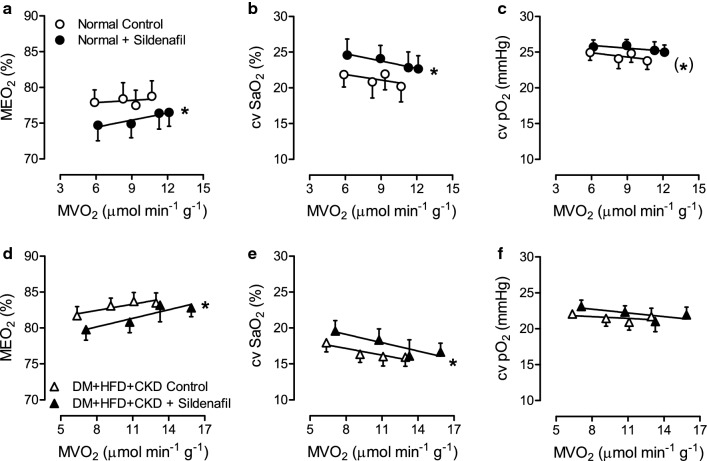
In vivo effect of PDE5 inhibition on myocardial oxygen balance at rest and during exercise in Normal and DM + HFD + CKD swine. Effect of phosphodiesterase 5 inhibition with Sildenafil during exercise on myocardial oxygen extraction (MEO_2_, **a** and **d**), on coronary venous oxygen saturation (cv SaO_2_, **b** and **e**) and coronary venous partial oxygen pressure (cv pO_2_, **c** and **f**) were not different between groups. Values are mean ± SEM. DM + HFD + CKD: *n* = 6, Normal: *n* = 7. *P < 0.05 and (*)*P* = 0.067 versus corresponding Control by two-way ANCOVA for repeated measures

To test whether the loss of endogenous coronary vasodilator influence of NO in DM + HFD + CKD swine was, at least in part, mediated by an increase in PDE5 activity thereby reducing cGMP levels, swine were subjected to exercise in the absence and presence of PDE5 inhibitor Sildenafil. Haemodynamic and myocardial oxygen balance data are shown in Table 3 and Fig. 6. Sildenafil produced modest reductions in mean aortic pressure that were slightly more pronounced in Normal than in DM + HFD + CKD swine (*P* = 0.047), and were accompanied by modest increases in heart rate that were not different between groups (Table 3).

**Table 3:** Haemodynamic and myocardial oxygen balance during exercise with and without PDE5 inhibition in Normal and DM + HFD + CKD swine

	Group	Intervention	*n*	Standing	Exercise			ANOVA *P* value		
					2	3	4	Sildenafil	Group	Sildenafil *Group
Heart rate (beats min^−1^)	Normal	Control	8	125 ± 8	174 ± 12*	207 ± 12*	226 ± 10*			
		Sildenafil	8	137 ± 4	195 ± 10*	211 ± 10*	232 ± 9*	0.076		
	DM + HFD + CKD	Control	6	124 ± 6	177 ± 14*	185 ± 14*	205 ± 13*		0.318	
		Sildenafil	6	143 ± 5^‡^	173 ± 7*	201 ± 8*	229 ± 11*	0.010	0.729	0.518
MAP (mmHg)	Normal	Control	8	90 ± 4	92 ± 3	95 ± 3	96 ± 3			
		Sildenafil	8	75 ± 2^‡^	82 ± 3*^‡^	89 ± 2*^‡^	90 ± 2*	< 0.001		
	DM + HFD + CKD	Control	6	87 ± 4	88 ± 5	89 ± 4	92 ± 5		0.018	
		Sildenafil	6	75 ± 5	83 ± 5	89 ± 5*	95 ± 5*	0.219	0.581	0.047
CBF (ml min^−1^ g^−1^)	Normal	Control	7	1.24 ± 0.18	1.57 ± 0.16	1.82 ± 0.13*	2.03 ± 0.17*			
		Sildenafil	7	1.28 ± 0.09	1.90 ± 0.13*	2.08 ± 0.18*	2.08 ± 0.20*	0.108		
	DM + HFD + CKD	Control	6	1.28 ± 0.13	1.82 ± 0.33	1.98 ± 0.33	2.34 ± 0.29*		0.314	
		Sildenafil	6	1.53 ± 0.21	2.00 ± 0.22	2.32 ± 0.28*	2.65 ± 0.27*	0.121	0.066	0.601
Hemoglobin (g dl^−1^)	Normal	Control	7	9.7 ± 0.5	11.1 ± 0.5	11.0 ± 0.9	11.2 ± 0.8			
		Sildenafil	7	10.7 ± 0.6	10.3 ± 0.7	11.5 ± 0.7	12.5 ± 0.8	0.359		
	DM + HFD + CKD	Control	6	9.5 ± 0.4	9.6 ± 0.4^†^	10.5 ± 0.6	10.4 ± 0.6		0.720	
		Sildenafil	6	9.6 ± 0.6	11.1 ± 0.6	11.6 ± 1.0	12.0 ± 0.9	0.037	0.679	0.445
Arterial SaO_2_ (%)	Normal	Control	7	99 ± 1	97 ± 1*	97 ± 1	95 ± 1			
		Sildenafil	7	97 ± 1^‡^	96 ± 1	97 ± 1	96 ± 1	0.083		
	DM + HFD + CKD	Control	6	98 ± 1	97 ± 1	99 ± 1	97 ± 1		0.574	
		Sildenafil	6	97 ± 1	96 ± 1	96 ± 1	97 ± 1	0.021	0.720	0.554
CV SaO_2_ (%)	Normal	Control	7	21.9 ± 1.75	20.8 ± 2.3	21.9 ± 2.2	20.2 ± 2.2			
		Sildenafil	7	24.6 ± 2.3	24.1 ± 1.8^‡^	22.8 ± 2.2	23.5 ± 1.8	0.242		
	DM + HFD + CKD	Control	6	18.0 ± 1.3	16.4 ± 1.1	16.0 ± 1.3^†^	15.9 ± 1.3		< 0.001	
		Sildenafil	6	19.6 ± 1.4	18.3 ± 1.5^†^	16.1 ± 2.3	16.6 ± 1.3^†^	0.220	< 0.001	0.367
CV pO_2_ (mmHg)	Normal	Control	7	25.0 ± 1.1	24.1 ± 1.4	24.8 ± 1.3	23.8 ± 1.2			
		Sildenafil	7	25.7 ± 1.0	25.9 ± 0.8	25.3 ± 1.2	25.8 ± 1.2	0.073		
	DM + HFD + CKD	Control	6	22.0 ± 0.4^†^	21.4 ± 1.1	20.9 ± 1.1^†^	21.7 ± 1.2		< 0.001	
		Sildenafil	6	23.1 ± 0.9	22.3 ± 0.9^†^	21.0 ± 1.4^†^	21.9 ± 1.1^†^	0.220	< 0.001	0.534
MVO_2_ (µmol min^−1^ g^−1^)	Normal	Control	7	5.9 ± 0.9	8.3 ± 0.7	9.3 ± 0.7*	10.7 ± 1.1*			
		Sildenafil	7	6.2 ± 0.3	8.9 ± 1.1*	11.3 ± 1.5*	12.3 ± 0.9*	0.164		
	DM + HFD + CKD	Control	6	6.4 ± 0.8	9.2 ± 1.9	11.1 ± 2.0	12.9 ± 2.1*		0.222	
		Sildenafil	6	7.4 ± 1.4	10.7 ± 1.2	13.3 ± 1.6*	15.9 ± 1.7*	0.117	0.020	0.414

Values are mean ± SEM

*MAP* mean arterial pressure, *CBF* coronary blood flow per gram of myocardium, *SaO*_*2*_ oxygen saturation, *CV* coronary venous, *pO*_*2*_ partial oxygen pressure, *MVO*_*2*_ myocardial oxygen consumption per gram of myocardium. The phosphodiesterase 5 inhibitor Sildenafil was administered in a dose of 10 mg (IV)

*P < 0.05 versus corresponding Standing

^†^*P* < 0.05 versus corresponding Normal

^‡^*P* < 0.05 versus corresponding Control by two-way ANOVA for repeated measures and post hoc analysis with least significant difference correction

PDE5 inhibition in Normal swine resulted in a small increase in myocardial oxygen delivery at each level of myocardial oxygen consumption, resulting in a decreased myocardial O_2_ extraction associated with an increase in coronary venous O_2_ saturation and a trend towards an increase in coronary venous O_2_ tension (Fig. 6a–c). Similar effects were observed in DM + HFD + CKD swine (Fig. 6d–f). These observations indicate that PDE5 inhibition resulted in a vasodilator response of coronary resistance vessels, and suggest that coronary microvascular PDE5 activity was not increased in DM + HFD + CKD swine. Indeed, analysis of left ventricular myocardial samples showed that overall PDE activity (6.7 ± 0.5 vs. 5.7 ± 0.4 AU µg protein^−1^, *P* = 0.18) and specifically PDE5 activity (1.58 ± 0.39 vs. 0.78 ± 0.46 AU µg protein^−1^, *P* = 0.20) as well as PDE5 mRNA expression (0.029 ± 0.002 vs. 0.027 ± 0.004 AU, *P* = 0.78), were not different between the Normal and DM + HFD + CKD swine, respectively.

### In vitro assessment of NO bioavailability

**Fig. 7:**
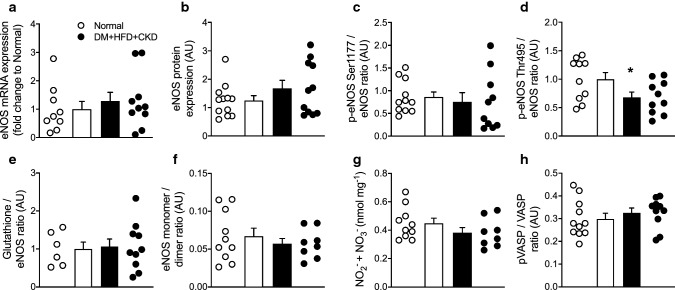
In vitro assessment of left ventricular endothelial nitric oxide synthase (eNOS) and NO bioavailability in Normal and DM + HFD + CKD swine. Left ventricular endothelial eNOS mRNA (**a**) and protein (**b**) levels, as well as phosphorylation (p-eNOS) at the Ser1177 site (**c**) were not different between groups, while p-eNOS at the inhibitory Thr495 site was lower in DM + HFD + CKD swine (**d**). No differences between groups in eNOS glutathionylation (**e**), coupling (**f**), myocardial nitric oxide metabolites nitrite (NO_2_^−^) and nitrate (NO_3_^−^, **g**) and the ratio of phosphorylated to non-phosphorylated vasodilator activated phosphoprotein (p-VASP/VASP, **h**) were observed. Values are mean ± SEM. The original western blots are available in the online supplemental file: original eNOS and p-eNOS (Ser1177) western blots are shown in Supplementary Fig. 6, p-eNOS (Thr495), VASP and p-VASP blots are shown in Supplementary Fig. 7, Glutathione/eNOS blots are shown in Supplementary Fig. 8, and eNOS monomer/dimer blots are shown in Supplementary Fig. 9. *P < 0.05 for DM + HFD + CKD versus Normal by Student’s *t* test

**Fig. 8:**
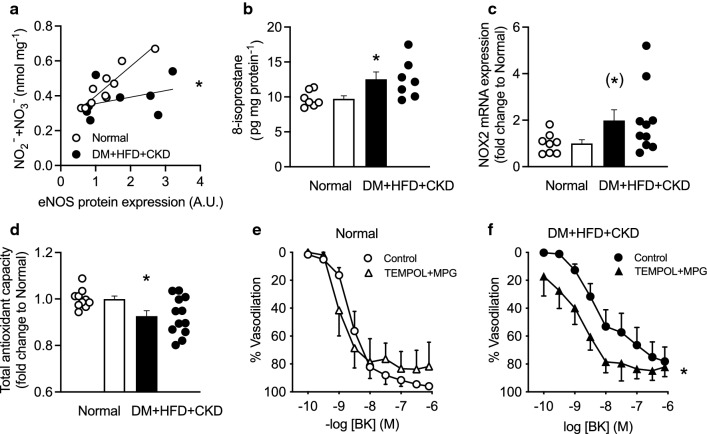
In vitro assessment of left ventricular NO bioavailability and oxidative stress in Normal and DM + HFD + CKD swine. Left ventricular myocardial nitrite (NO_2_^−^) and nitrate (NO_3_^−^) levels plotted as a function of eNOS protein levels (**a**), left ventricular 8-isoprostane levels (**b**), NADPH-oxidase 2 (NOX2) mRNA expression levels (**c**) left ventricular total antioxidant capacity (**d**), and in vitro small coronary artery endothelium-dependent vasodilation to bradykinin (BK) in the absence/presence of ROS scavengers Tempol and MPG (**e** and **f**) in Normal and DM + HFD + CKD swine. Values are mean ± SEM. *P < 0.05 and (*)*P* = 0.086 for DM + HFD + CKD versus Normal by logistic regression analysis (**a**), Student’s t test (**b–d** ) and two-way ANOVA for repeated measures (**f**)

NO production, bioavailability, and signalling were studied in bulk LV myocardial samples. mRNA and protein levels of eNOS, eNOS phosphorylation at the stimulatory Ser1177 site, eNOS glutathionylation and eNOS monomer to dimer ratio were all maintained, while eNOS phosphorylation at the inhibitory Thr495 site was even slightly lower in DM + HFD + CKD swine (Fig. 7a–f). Accordingly, concentrations of NO metabolites NO_2_^−^ + NO_3_^−^ and the p-VASP/VASP ratio were also not significantly different between groups (Fig. 7g, h), suggesting that NO production and signalling was maintained in bulk myocardial tissue. Similar to the myocardial levels, plasma NO_2_^−^ + NO_3_^−^ levels were not different between groups (Normal 3.2 ± 0.7 vs. DM + HFD + CKD 4.4 ± 1.0 µmol L^−1^, *P* = 0.3). Interestingly, however, DM + HFD + CKD swine showed a clockwise rotation in the relationship between eNOS protein levels and myocardial NO_2_^−^ + NO_3_^−^ levels as compared to Normal swine, so that at a given level of eNOS protein expression, the myocardial NO_2_^−^ + NO_3_^−^ levels were lower (Fig. 8a). Furthermore, myocardial 8-isoprostane levels were significantly increased (Fig. 8b), with trends towards an increase in myocardial NOX2 expression (Fig. 8c) and sNOX-dp in plasma (Supplementary Fig. 10), and a significantly lower total anti-oxidant capacity in DM + HFD + CKD swine (Fig. 8d). These observations suggest that the lower NO bioavailability may have been the result of scavenging of NO by reactive oxygen species. This hypothesis is supported by in vitro vascular functional data, showing that incubation with the free radical scavengers MPG and superoxide dismutase mimetic Tempol had no effect on bradykinin-induced vasodilation of isolated healthy coronary small arteries (Fig. 8e), but restored vasodilation to bradykinin in coronary small arteries isolated from DM + HFD + CKD swine (Fig. 8f).

## Discussion

The present study investigated the involvement of the NO pathway in the perturbations in myocardial oxygen delivery and oxygen balance in swine exposed to DM + HFD + CKD for 5 months. The main findings were that: (i) Endothelium-dependent vasodilator function of coronary resistance vessels was impaired in DM + HFD + CKD compared to Normal swine, both in vivo and in vitro. (ii) A loss of NO bioavailability was responsible for the blunted in vitro bradykinin-induced dilation of coronary small arteries of DM + HFD + CKD swine. (iii) The impairments in myocardial oxygen delivery and oxygen balance that were observed in DM + HFD + CKD swine at rest and during exercise, were also principally due to loss of NO bioavailability, with no change in PDE5 activity. (iv) The loss of NO bioavailability was likely due to increased oxidative stress, as myocardial lipid peroxidation was increased resulting in a decrease in overall anti-oxidant capacity, while in vitro treatment with antioxidants restored endothelial-dependent vasodilation of small arteries. The implications of these findings will be discussed below.

The pathophysiology of INOCA—previously named ‘cardiac syndrome-X’—remains incompletely understood, but coronary microvascular dysfunction is thought to play a pivotal role [1, 45]. Indeed, INOCA patients are often characterized by a reduced CFR, which is a strong predictor of cardiac mortality [1, 10, 55]. In agreement with those observations, we recently demonstrated in swine that the combination of DM, HFD and CKD resulted in a phenotype resembling INOCA, showing not only reduced CFR but also impaired myocardial perfusion and oxygen delivery, and signs of anaerobic cardiac metabolism [71]. These abnormalities were associated with reduced myocardial capillary density, increased interstitial fibrosis, and left ventricular diastolic dysfunction [60], but occurred in the absence of epicardial coronary atherosclerosis, consistent with the presence of coronary microvascular dysfunction. The latter did not appear to be due to structural abnormalities of coronary resistance vessels, as arteriolar density and geometry were unchanged, suggesting that impaired regulation of coronary microvascular tone was principally responsible for coronary microvascular dysfunction, possibly as a result of endothelial dysfunction [71].

There is increasing evidence from clinical studies that impaired microvascular endothelial dysfunction plays an important role in INOCA [1, 4, 10, 46, 55]. The results of the present study in swine are consistent with these previous reports by demonstrating coronary microvascular endothelial dysfunction induced by prolonged exposure to common risk factors. Thus, the combination of DM, HFD and CKD resulted in marked blunting of endothelium-dependent vasodilator responses to either ATP in vivo or bradykinin in vitro, particularly when expressed as a function of endothelium-independent vasodilator responses to SNP in vivo or SNAP in vitro, respectively.

In small arteries in vitro, the vasodilator response to endothelium-dependent vasodilator bradykinin was reduced, while the vasodilator response to the NO-donor SNAP was maintained. In contrast, in resting swine in vivo, the blunted vasodilator response to ATP (which in doses below 300 µg kg^−1^ min^−1^—as used in the present study—is completely blocked by NLA demonstrating that it is principally NO mediated [16]) was accompanied by an increased vasodilator response to NO-donor SNP. These findings suggest that coronary microvascular smooth muscle sensitivity to NO was increased thereby compensating for the blunted vasodilator response to ATP, possibly via increased responsiveness of soluble guanylyl cyclase (sGC) [41]. The difference between the in vitro response to SNAP and the in vivo response to SNP is not readily explained, but may be related to (i) differences in vessel segment, with small arteries in vitro versus a combination of small arteries and arterioles in vivo [9], and (ii) in vivo versus in vitro conditions, i.e. presence and absence of flow and shear stress [15], lower versus higher levels of oxygen and reactive oxygen species, and presence versus absence of surrounding myocardium [62], respectively. Most importantly, however, both in vitro and in vivo experiments demonstrated a relative blunting of the endothelium-dependent as compared to the endothelium-independent vasodilator responses.

Further examination of coronary small arteries in vitro showed that the vasodilator response to bradykinin in Normal swine was markedly attenuated by NO synthase inhibition with LNAME. In contrast, LNAME had no effect on the blunted vasodilator response to bradykinin in DM + HFD + CKD, indicating that the blunted coronary small artery dilator response to bradykinin in DM + HFD + CKD versus Normal swine was due to loss of NO bioavailability. Coronary microvascular dysfunction with loss of endothelium-dependent vasodilation has been previously described in coronary microvessels isolated from swine with metabolic derangement with [54, 60] or without [72] CKD, or familial hypercholesterolemia [2], from obese rats [30] and also from DM patients [7]. Although the exposure time, type and combination of risk factors varied between studies, the common denominator was the decrease in endothelium-dependent vasodilation, while the degree of dysfunction depended on the synergistic effect of the risk factors, as swine subjected to a combination (HFD and hypertension), had more pronounced attenuation of endothelium-dependent vasodilation, than those with only one risk factor [54]. Consistent with our findings, in the majority of these studies the underlying mechanism was loss of NO bioavailability [7, 30, 54, 60, 72], although loss of endothelium-derived hyperpolarizing factor with maintained NO-mediated vasodilation has also been reported [2]. Importantly, endothelial dysfunction (and its underlying mechanisms) appears to be a dynamic process during disease progression, as evidenced by two studies employing the same model of DM and hypercholesterolemia, demonstrating a shift from early (2.5 months) loss of NO-mediated vasodilation [72] to a late (15 months) normalization of NO signalling but increased vasoconstriction to endothelin-1 [64].

Consistent with our in vitro observations, NOS inhibition produced vasoconstriction of coronary resistance vessels in exercising Normal swine that was abolished in DM + HFD + CKD swine, indicating a loss of the endogenous NO-mediated coronary vasodilator influence. The observed reduction in coronary microvascular NO bioavailability could result from a decrease in NO production and/or an increase in NO degradation [6]. A decrease in NO production by microvascular eNOS can be caused by reduced eNOS protein levels or impaired eNOS function, due to reduced phosphorylation at the stimulatory Ser1177 site, increased phosphorylation at the inhibitory Thr495 site, or eNOS-uncoupling [6]. In the present study, total eNOS content, coupling status and phosphorylation at the Ser1177 site were all maintained, while Thr495 phosphorylation was even reduced. Consistent with these findings—the absolute NO_2_^−^ + NO_3_^−^ levels as measured in left ventricular myocardial bulk homogenates were also maintained in DM + HFD + CKD compared to Normal swine. Although it could be argued that the sensitivity of the Griess assay used to assess NO metabolites levels may not be sufficient to detect subtle differences in levels of NO metabolites [51], it should be noted that the ratio between p-VASP and VASP was also not different between the two groups, further suggesting that NO-cGMP signalling was maintained in bulk myocardial samples from DM + HFD + CKD swine.

An explanation for the lack of detectable changes in eNOS protein levels and function and NO metabolite levels in myocardial tissue samples in the presence of a marked loss of NO-mediated vasodilator influence in coronary resistance vessels is not readily found, but could be several-fold. For example, it cannot be excluded that a selective loss of NO in coronary microvessels remained undetected in bulk myocardial tissue samples due to predominance of cardiomyocytes and fibroblasts. Alternatively, it is possible that the reduced NO-mediated coronary vasodilator influence in DM + HFD + CKD swine was due to a loss of circulating NO. Thus, not only vascular endothelium but also red blood cells express eNOS [35], which has been shown to contribute to the regulation of blood flow either by influencing vascular tone or red blood cell deformability, and hence microcirculatory resistance to blood flow [28, 75]. Moreover, impaired red blood cell deformability has been shown to be present in patients with diabetes mellitus and coronary artery disease [33], which could have contributed to the perturbations in coronary vascular resistance and myocardial oxygen delivery in our DM + HFD + CKD swine. In the present study, we did not determine the exact contribution of a loss of circulating NO to the coronary microvascular dysfunction. However, the lack of a decrease in plasma NO_2_^−^ + NO_3_^−^ in conjunction with the loss of NO-mediated vasodilation by bradykinin in isolated buffer-perfused coronary small arteries, suggests that the loss of NO-mediated vasodilator influence occurred principally in the coronary microvascular endothelium.

Interestingly, a clockwise rotation in the relationship between NO_2_^−^ + NO_3_^−^ levels and eNOS protein content was observed in the DM + HFD + CKD compared to the Normal group (Fig. 7a), suggesting that NO bioavailability was impaired at a given level of eNOS in DM + HFD + CKD animals. NO bioavailability is not only determined by NO production, but also by its half-life. For example, albumin can bind NO and prolong its half-life. Hypoalbuminemia, which is a marker of cardiovascular disease in patients with CKD [32, 56], and which was also observed in our DM + HFD + CKD swine, may result in a reduction of circulating albumin-bound NO, thereby shortening the half-life of produced NO and impairing NO-mediated vasodilation [38, 65]. In the present study, we did not investigate the possible contribution of hypoalbuminemia to the reduced bioavailability of NO, but again the maintained levels of plasma NO_2_^−^ + NO_3_^−^, together with the loss of bradykinin-induced NO-mediated vasodilation in buffer-perfused coronary small arteries, suggests that the loss of NO-mediated vasodilator influence occurred principally in the coronary microvascular endothelium.

The loss of NO bioavailability and its vasodilator influence in the coronary microvasculature could also be the result of NO scavenging by reactive oxygen species, due to increased vascular oxidative stress [6, 67]. Indeed, we previously observed increased myocardial oxidative stress in this model, with an increase in the production of superoxide anions, that was positively correlated with the increase in TNF-α [60]. TNF-α is an important mediator of inflammation that has not only been shown to contribute to myocardial contractile dysfunction [14], but which was also shown to produce a pro-inflammatory state in isolated coronary arterioles, that attenuated NO-mediated vasodilation, and which was restored by reactive oxygen species scavenging [76]. Such effects were not only confined to TNF-α as C-reactive protein was also shown to induce oxidative stress thereby limiting NO-dependent endothelial vasodilation in coronary arterioles [50]. These findings suggest that inflammation in general is able to induce a pro-oxidative state in the coronary microcirculation thereby attenuating NO-mediated vasodilation. Such a pro-oxidative state was confirmed in the present study, as the endothelium-dependent vasodilation of isolated coronary small arteries of DM + HFD + CKD swine in response to bradykinin normalized in the presence of antioxidant treatment with MPG and Tempol. In this respect, it should be noted that our study was performed in adolescent swine. Since older age is associated with vascular and immune cell senescence [31], it could be speculated that the coronary vascular phenotype would be more pronounced at an advanced age. Indeed, middle-aged patients with microvascular angina showed a more than 50% decrease in total antioxidant capacity [34], as compared to a 22% decrease that we observed in adolescent DM + HFD + CKD swine. Different mechanisms, including xanthine oxidase [76] and NOX [50] and eNOS uncoupling [60], as well as increased myocardial lipid peroxidation have been proposed to be responsible for this increased oxidative stress. Indeed, significantly higher myocardial 8-isoprostane levels were observed in the present study, which were accompanied by a doubling of NOX2 expression in bulk myocardium of swine with DM + HFD + CKD that reached borderline statistical significance (*P* = 0.08). Importantly, in the present study, the endothelium-dependent vasodilation of isolated coronary small arteries to bradykinin was restored to Normal levels by antioxidant treatment with MPG and Tempol. An increase in oxidative stress with a reduction in NO-mediated vasodilation has also been demonstrated in the peripheral circulation of patients with DM [23, 25] and hypercholesterolemia [24], which was improved by antioxidant or tetrahydrobiopterin (eNOS-cofactor) treatment. Additionally, antioxidant supplementation had beneficial effects of on endothelial function in patients with chronic kidney disease [11, 19, 43]. Future studies are required to further explore the specific mechanisms underlying the alterations in nitroso-redox balance in the presence of risk factors and their influence on the regulation of coronary microvascular tone.

The vasodilator influence of NO not only depends on its bioavailability but also on the half-life of its second messenger cGMP, which is regulated by degradation of cGMP through PDE5. Previous studies from our laboratory have shown alterations in the microvascular constrictor influence of PDE5 in the coronary [37] and pulmonary [73] vascular beds of swine with a chronic myocardial infarction, indicating that changes in PDE5 activity can contribute to microvascular dysfunction. Consequently, we investigated the contribution of PDE5 to the regulation of coronary microvascular tone in our swine model of INOCA. The vasoconstrictor influence of PDE5 did not appear to be altered by the risk factors, as neither the vasodilator responses to sildenafil, nor myocardial PDE5 expression and activity were different between groups. Taken together, these findings suggest that PDE5 did not contribute to the blunted vasodilator influence of endogenous NO in DM + HFD + CKD swine.

There are unfortunately no other studies available on the contribution of PDE5 to coronary microvascular dysfunction in experimental models of INOCA, and the few available clinical studies show mixed results. Thus, an early study in patients with angina and no, angiographically visible, obstructive coronary artery disease, reported that 100 mg PO Sildenafil—a dose that dilated proximal epicardial coronary artery segments and produced a small decrease in arterial blood pressure—had no effect on basal coronary vascular resistance and no effect on the acetylcholine-induced, endothelium-dependent reduction in coronary resistance [22]. In contrast, in the WISE study, Sildenafil—also in a dose of 100 mg PO—increased CFR in women with microvascular angina without obstructive coronary artery disease and a CFR < 2.5, with the effect being largest in patients with the lowest baseline CFR [13]. Taken together, PDE5 inhibition has a limited vasodilator effect in the coronary microcirculation. However, data with respect to the coronary vasodilator effect of PDE5 inhibition in the setting of INOCA are ambiguous and require further investigation.

In addition to its role in regulating coronary microvascular tone, NO can also act as a paracrine signalling molecule on myocardial function and metabolism [27, 49, 68]. Indeed several studies, have shown that inhibition of NO synthase results in increases in myocardial oxygen consumption [59, 68], shifts in substrate utilization [3, 36] and reduced contractile function [27, 52] not only in the healthy heart [3, 27, 36, 52, 59, 68] but also during moderate myocardial ischemia [27, 36], or in pacing-induced congestive heart failure [53, 68]. We therefore hypothesized that the increase in myocardial oxygen consumption at a given level of rate-pressure product in DM + HFD + CKD swine could be explained by the loss of NO signalling, similar to what was reported in awake dogs following chronic NO synthase inhibition [49]. However, yet in accordance with our previous observations [16], we failed to observe changes in LV function and myocardial oxygen consumption in response to acute inhibition of NO synthase in either normal or DM + HFD + CKD swine either at rest or during exercise (Supplementary Figs. 4 and 5). Although it cannot be excluded that a chronic loss of NO may exert effects that are different from those exerted by acute pharmacological NOS inhibition, these findings do not support the concept that a loss of NO contributed to the increase in myocardial oxygen consumption at a given level of RPP in the DM + HFD + CKD swine. Future studies are required to further investigate the mechanisms underlying the increased myocardial oxygen consumption in DM + HFD + CKD swine.

In the present study in swine, we used a combined in vitro and in vivo approach to investigate coronary microvascular function in this complex animal model with multiple risk factors. Although very valuable mechanistically [5, 39], in vitro testing of coronary small arteries of patients is difficult since vascular samples are very scarce and typically restricted to atrial vessels obtained during coronary artery bypass grafting or valve surgery. In the clinical setting, in vivo coronary microvascular function is typically assessed by invasive pharmacological coronary vasomotor function tests, using thermodilution (index of microcirculatory resistance) or flow wire (CFR). A limitation of the flow wire is that it measures velocities, rather than volumetric flow. More recently, cardiac positron emission tomography and MRI are becoming additional non-invasive tools that are able to measure blood flow per gram of myocardium showing in INOCA patients that—similar to the observations in the present study in swine—myocardial perfusion reserve is reduced [1, 17]. Although these methods have been shown to be valuable diagnostic and prognostic tools for coronary microvascular dysfunction in INOCA [1, 47, 71], they have not been applied in studies of the dynamics of coronary blood flow regulation in response to physiological stimuli, such as physical exercise, in patients. In the present study, we, therefore, set out to investigate the coronary blood flow responses to exercise in chronically instrumented swine. Since changes in coronary blood flow are tightly coupled to myocardial oxygen demand, we investigated the myocardial oxygen balance, correlating myocardial oxygen delivery to myocardial oxygen demand [70]. Plotting myocardial oxygen extraction, and coronary venous oxygen tension and saturation as a function of either myocardial oxygen consumption (Fig. 5) or the rate-pressure-product (Supplementary Fig. 2) allows sensitive assessment of coronary resistance vessel tone independent of changes in myocardial oxygen demand [15]. While such measurements have occasionally also been performed in humans, providing valuable insights in resistance vessel tone regulation in the human heart [15], it requires catheterization of the great cardiac vein, limiting its current use in the clinical setting.

## Conclusions

**Fig. 9:**
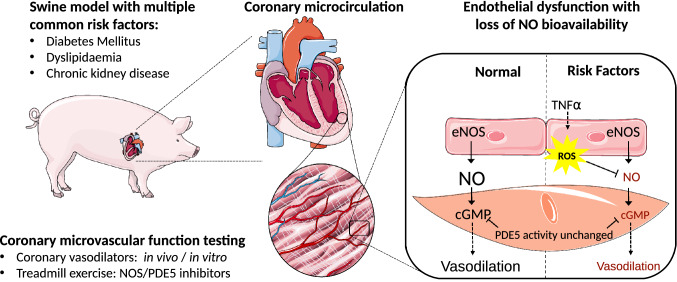
Proposed mechanisms contributing to coronary microvascular dysfunction in DM + HFD + CKD swine. NOS, nitric oxide synthase; PDE5, phosphodiesterase 5; NO, nitric oxide, eNOS, endothelial nitric oxide synthase; cGMP, cyclic guanosine monophosphate; TNFα, tumor necrosis factor α; ROS, reactive oxygen species

Recently, we reported that prolonged exposure of swine to DM + HFD + CKD leads to reduced CFR and to perturbations in myocardial oxygen balance during treadmill exercise [71]. The present study demonstrates that the perturbations in myocardial oxygen balance are associated with coronary microvascular endothelial dysfunction, and are the result of a loss of NO-mediated vasodilator influence. The latter is not due to an increased PDE5 activity but appears to be the result of reduced NO bioavailability, likely due to increased oxidative stress, as myocardial lipid peroxidation was increased resulting in a decrease in overall anti-oxidant capacity, while in vitro treatment with antioxidants restored endothelial-dependent vasodilation of small arteries. Figure 9 summarizes the proposed mechanisms contributing to coronary microvascular dysfunction in DM + HFD + CKD swine. Future studies are required to investigate the potential benefit of improving NO bioavailability and NO-cGMP signalling in vivo and to determine whether this will normalize microvascular function and restore myocardial oxygen balance in this translational large animal model of coronary microvascular dysfunction.

## Supplementary Information

Below is the link to the electronic supplementary material.

Supplementary file1 (DOCX 3616 KB)

## Data Availability

All relevant data are within the paper and its Supporting Information files.
