# Supplementary material for: Reduced nitric oxide bioavailability impairs myocardial oxygen balance during exercise in swine with multiple risk factors
Source: Basic Res Cardiol. 2021 Aug 26;116(1):50. doi: 10.1007/s00395-021-00890-8 (PMC8387273; doi:10.1007/s00395-021-00890-8)
Supplement: Supplementary file 1 — Supplementary file1 (DOCX 3616 KB) [file 395_2021_890_MOESM1_ESM.docx]

**Reduced Nitric Oxide Bioavailability Impairs Myocardial Oxygen Balance During Exercise in Swine with Multiple Risk Factors**

*Basic Research in Cardiology*

Jens van de Wouw^1^ MD, MSc, Oana Sorop^1^ PhD, Ruben W.A. van Drie^1^ BSc, Jaap A. Joles^2^ DVM, PhD, A.H. Jan Danser^3^ PhD, Marianne C. Verhaar^2^ MD, PhD, Daphne Merkus^1,4,5^ PhD and Dirk J. Duncker^1^ MD, PhD

^1^Division of Experimental Cardiology, Department of Cardiology, Erasmus University Medical Center, Rotterdam, ^2^Department of Nephrology and Hypertension, University Medical Center Utrecht, Utrecht, ^3^Department of Internal Medicine, Erasmus MC University Medical Center, Rotterdam, Netherlands, ^4^Walter Brendel Center of Experimental Medicine (WBex), LMU Munich, 81377, Munich, Germany, ^5^German Center for Cardiovascular Research (DZHK), Partner Site Munich, Munich Heart Alliance (MHA), 81377, Munich, Germany

Corresponding author: Dirk J. Duncker, MD, PhD

Division of Experimental Cardiology

Department of Cardiology, Thoraxcenter

Erasmus University Medical Center,

PO Box 2040, 3000 CA Rotterdam

The Netherlands

Telephone: +31 10 7038066

E-mail: [d.duncker@erasmusmc.nl](mailto:d.duncker@erasmusmc.nl)

| **Supplemental Table 1.** Primer Sequences used for qPCR | | |
| --- | --- | --- |
| Gene | Forward sequence | Backward sequence |
| NOS3 | GGACACACGGCTAGAAGAGC | TCCGTTTGGGGCTGAAGATG |
| NOX2 | CCGCATTGTTGGCGACTGGA | CCCGTCCACAGCGATCTTAGG |
| PDE5A | GCCACTCAATCATGGAGCATC | GGAGAGGCCACTGAGAATCTG |
| HPRT1 | GGACTTGAATCATGTTTGTG | CAGATGTTTCCAAACTCAAC |
| RPL13A | TGGCCAAGCAGGTACTTCTG | GTATTCATGCGCTTGCGGAG |
| NOS3 endothelial nitric oxide synthase, NOX2 nicotinamide adenine dinucleotide phosphate (NADPH) oxidase 2, PDE5A phosphodiesterase 5, HPRT1 hypoxanthine phosphoribosyltransferase 1, RPL13A ribosomal protein L13a. | | |

| **Supplemental Table 2.** Systemic hemodynamic responses to SNP and ATP infusion in Normal and DM+HFD+CKD swine | | | | | | | | | | | | | | | | | | | | | | | | | | | | | | | | | | |
| --- | --- | --- | --- | --- | --- | --- | --- | --- | --- | --- | --- | --- | --- | --- | --- | --- | --- | --- | --- | --- | --- | --- | --- | --- | --- | --- | --- | --- | --- | --- | --- | --- | --- | --- |
|  |  | | Standing | | | | SNP (µg kg^-1^ min^-1^) | | | | | | | | | | | | | | | | | | | | | | | | | | | |
|  |  | n |  | | | | 0.5 | | | | 1 | | | | | 2 | | | | 3 | | | | | 4 | | | | | 5 | | | | |
| Heart rate | Normal | 9 | 102 | ± | | 6 | 105 | ± | 3 | | 104 | | ± | 4 | | 114 | ± | 6 | | 115 | | ± | 5 | | 123 | | ± | | 8* | 126 | | ± | | 8* |
| (beats min^-1^) | DM+HFD+CKD | 6 | 84 | ± | | 4† | 97 | ± | 6 | | 121 | | ± | 7*† | | 114 | ± | 4* | | 124 | | ± | 18 | | 136 | | ± | | 10* | 130 | | ± | | 10* |
| MAP | Normal | 9 | 95 | ± | | 2 | 90 | ± | 4 | | 90 | | ± | 3 | | 83 | ± | 3* | | 76 | | ± | 4* | | 81 | | ± | | 3* | 80 | | ± | | 4* |
| (mmHg) | DM+HFD+CKD | 6 | 92 | ± | | 8 | 79 | ± | 5 | | 60 | | ± | 7*† | | 58 | ± | 7*† | | 60 | | ± | 6* | | 63 | | ± | | 8* | 58 | | ± | | 4*† |
|  | | | | | | | | | | | | | | | | | | | | | | | | | | | | | | | | | | |
|  |  |  | Standing | | | | ATP (µg kg^-1^ min^-1^) | | | | | | | | | | | | | | | | | | | | | | | | | | | |
|  |  | n |  | | | | 50 | | | | | | | | 100 | | | | | | 200 | | | | | | | 300 | | | | | | |
| Heart rate | Normal | 8 | 96 | ± | 6 | | 97 | | | ± | | 4 | | | 105 | | ± | | 7 | | 122 | | | ± | | 8* | | 127 | | | ± | | 5* | |
| (beats min^-1^) | DM+HFD+CKD | 6 | 81 | ± | 5 | | 85 | | | ± | | 7 | | | 85 | | ± | | 5 | | 101 | | | ± | | 7 | | 115 | | | ± | | 10* | |
| MAP | Normal | 8 | 103 | ± | 3 | | 100 | | | ± | | 4 | | | 93 | | ± | | 5 | | 77 | | | ± | | 6* | | 66 | | | ± | | 4* | |
| (mmHg) | DM+HFD+CKD | 6 | 106 | ± | 7 | | 98 | | | ± | | 6 | | | 97 | | ± | | 7 | | 81 | | | ± | | 6* | | 69 | | | ± | | 8* | |
| Heart rate and mean arterial pressure (MAP) of Normal and DM+HFD+CKD during graded infusion with sodium nitroprusside (SNP) or adenosine triphosphate (ATP). From 2 µg kg^-1^ min^-1^ till 4 µg kg^-1^ min^-1^ SNP we had to discontinue the experiment in 1 animal at every dose due to dangerously low mean arterial pressures (<40 mmHg), Values are mean±SEM. *P<0.05 versus standing within group; †P<0.05 versus corresponding Normal by two-way ANCOVA for repeated measures. | | | | | | | | | | | | | | | | | | | | | | | | | | | | | | | | | | |

**Supplemental Figure 1.** Experimental timeline of Normal and DM+HFD+CKD swine

Experimental timeline of Normal and DM+HFD+CKD from risk factor induction to the termination. Normal swine were weight- and age-matched to the experimental group, did not have any risk factors induced and were fed normal chow. Normal swine underwent chronic instrumentation and *in vivo* experiments and termination according to a similar protocol as the experimental swine. The in vivo exercise experiments were performed in random order. w week, d day, DM diabetes mellitus, post-op post-operation, CKD chronic kidney disease, GFR glomerular filtration rate, CFR coronary flow reserve, PDE5i phosphodiesterase 5 inhibition, NLA N_ω_-Nitro-L-arginine, SNP sodium nitroprusside, ATP adenosine triphosphate.

**Supplemental Figure 2.** *In vivo* effect of NOS inhibition during exercise in Normal and DM+HFD+CKD swine

**The relationship between the rate pressure product (RPP, the product of heart rate and systolic arterial pressure) and myocardial oxygen extraction (MEO_2_, **a**&**d**), coronary venous oxygen saturation (cv SaO_2_, **b**&**e**), partial oxygen pressure (cv pO_2_, **c**&**f**) in Normal and DM+HFD+CKD swine at rest and during exercise, in control conditions and in the presence of NO synthase inhibition with N_ω_-Nitro-L-arginine (NLA). Values are mean ± SEM. DM+HFD+CKD: n=6, Normal: n=7. *P<0.05 versus corresponding Normal by two-way ANCOVA for repeated measures.

**Supplemental Figure 3.** *In vivo* effect of NOS inhibition during exercise in Normal and DM+HFD+CKD swine

Myocardial oxygen extraction (MEO_2_, **a**) was significantly lower and coronary venous oxygen saturation (cv SaO_2_,**b**) and partial oxygen pressure (cv pO_2_, **c**) were significantly higher in healthy Normal swine than in DM+HFD+CKD swine. These differences disappeared eNOS was inhibited by N_ω_-Nitro-L-arginine (NLA) during exercise (**d-f**). Values are mean ± SEM. DM+HFD+CKD: n=6, Normal: n=7. *P<0.05 versus corresponding Normal by two-way ANCOVA for repeated measures.

**Supplemental Figure 4**

The relationship between myocardial O_2_ consumption (MVO_2_), and rate pressure product (RPP) was not affected by the acute inhibition of NO synthase by N_ω_-Nitro-L-arginine (NLA) in either Normal (**a**) or DM+HFD+CKD (**b**) swine either at rest or during exercise. Values are mean ± SEM. DM+HFD+CKD: n=6, Normal: n=7.

**Supplemental Figure 5**

Maximum rate of rise of left ventricular pressure (LV dP/dt_max_) plotted as a function of the product of heart rate (HR) and diastolic arterial pressure (DAP) – two major determinants of myocardial oxygen consumption – in Normal (**a**) and DM+HFD+CKD (**b**) swine, in control conditions and in the presence of NO synthase inhibition with N_ω_-Nitro-L-arginine (NLA) at rest or during exercise. Values are mean ± SEM. Normal: n=4, DM+HFD+CKD: n=3.

**Supplemental Figure 6.** Western blot stained for phosphorylated eNOS at the Ser1177 site, total eNOS and β-actin.


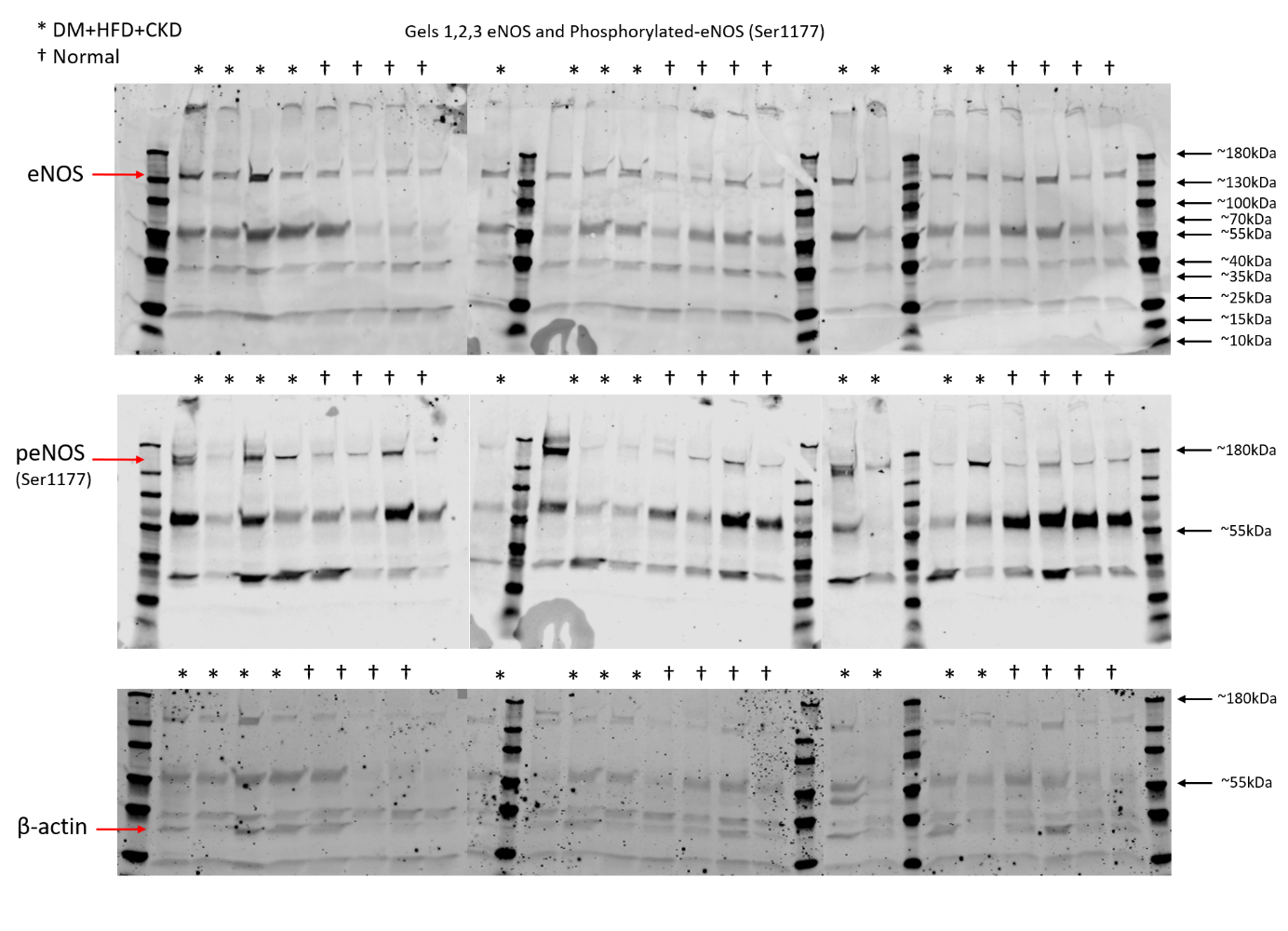


**Supplemental Figure 7.** Western blot stained for phosphorylated eNOS at the Thr495 site, phosphorylated VASP, total eNOS and total VASP.


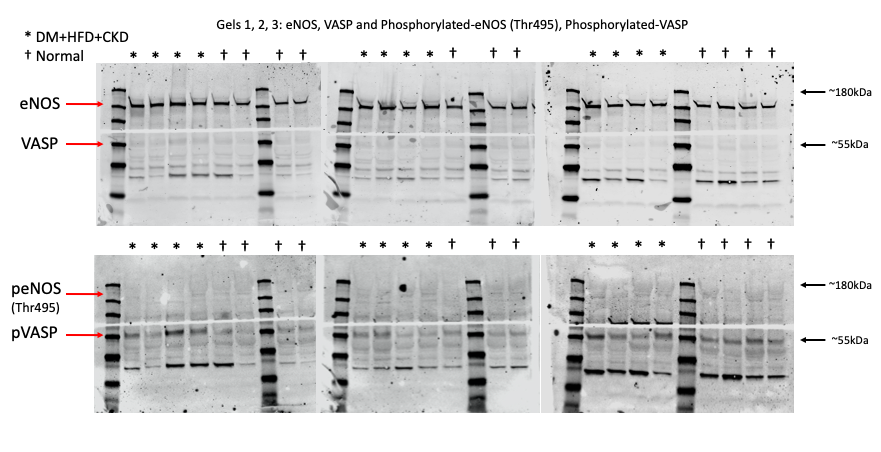


**Supplemental Figure 8.** Western blot stained for Glutathione and total eNOS after immunoprecipitation for eNOS.


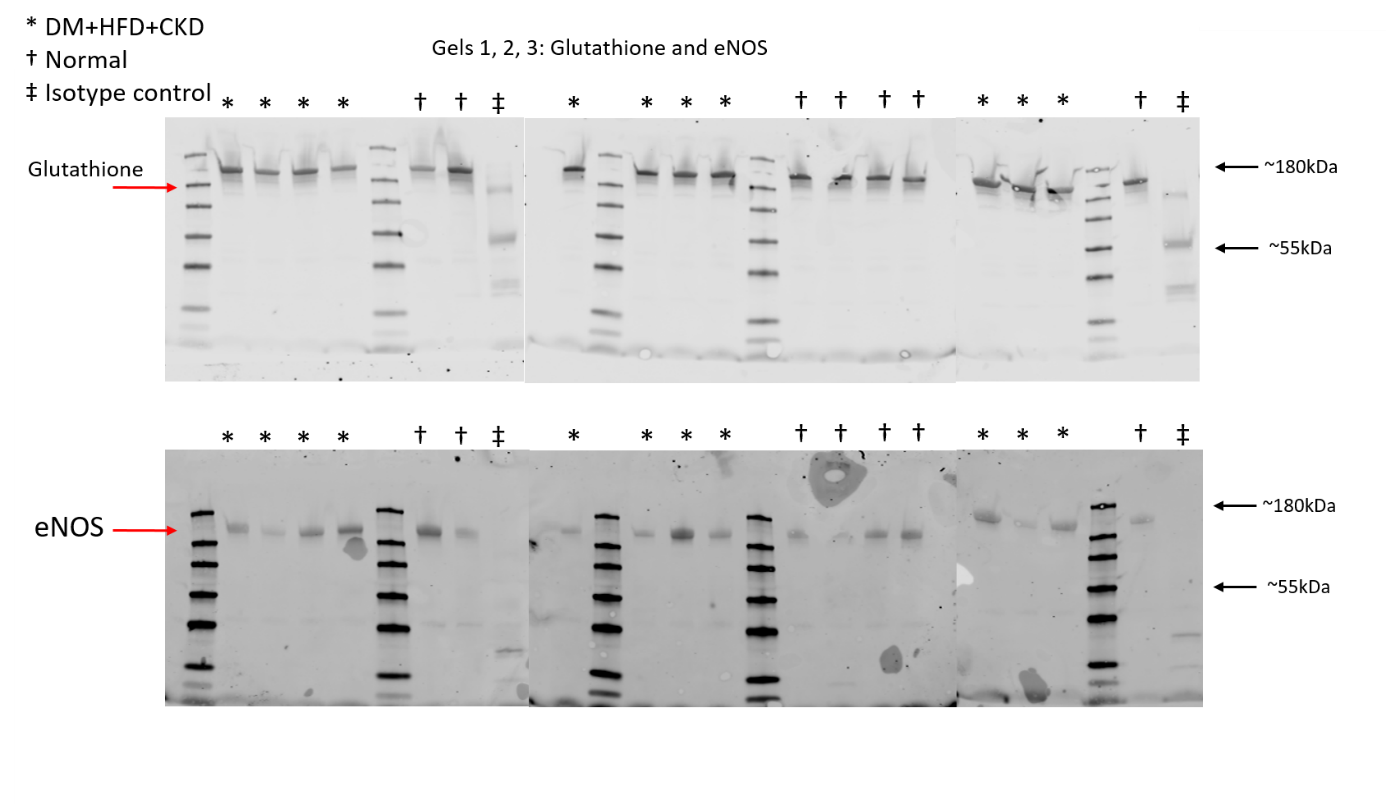


**Supplemental Figure 9.** Western blot stained for monomer and dimer of eNOS.


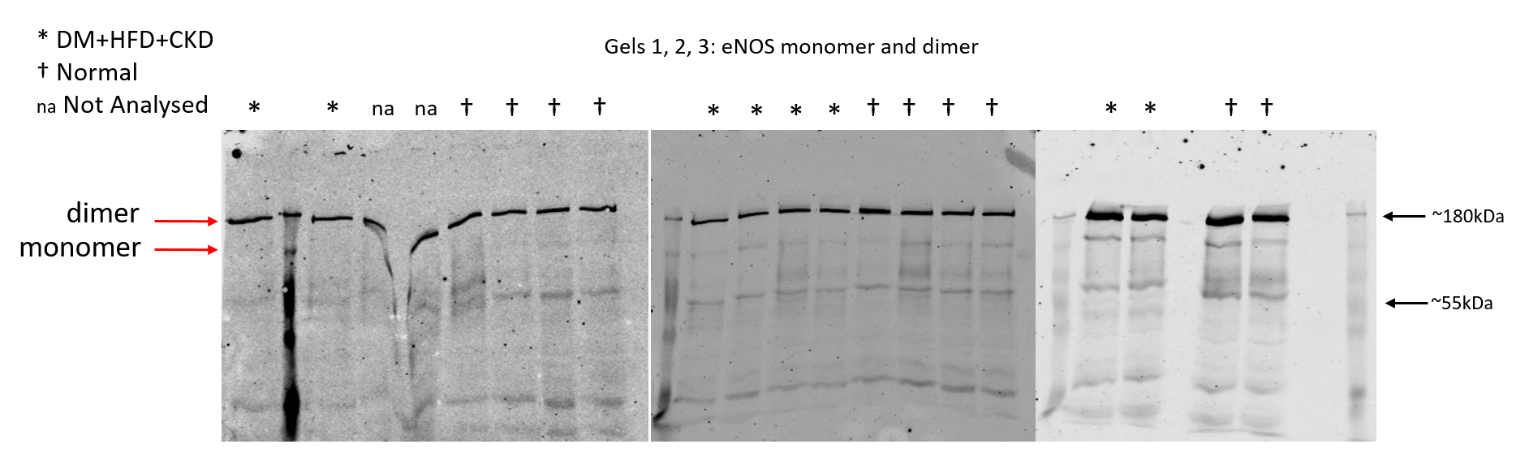


**Supplemental Figure 10.** NOX2-dp measurements in coronary venous plasma of DM+HFD+CKD and Normal animals.

sNOX2-dp measurement (E13651327, Sincere Bio, Beijing, China) was performed in undiluted coronary venous plasma of both Healthy and DM+HFD+CKD animals. Possibly due to the low cross-reactivity of the human antibody with the swine NOX-2 dp peptide – the measured values were very low despite using undiluted plasma (panels **a**&**b**), resulting in values below the lower detection limit of the kit (dotted line in panels **b**&**c**) in several of the animals (panel **c**). Additionally, the remaining detectable values were below the lowest value of the standard calibration curve (dashed line in panels **b**&**c**) provided by the kit (panels **a**&**b**). Notwithstanding these methodological limitations, we observed a small trend towards an increase in sdNOX2-dp in DM+HFD+CKD animals.
